# Community-based intermittent mass testing and treatment for malaria in an area of high transmission intensity, western Kenya: study design and methodology for a cluster randomized controlled trial

**DOI:** 10.1186/s12936-017-1883-z

**Published:** 2017-06-07

**Authors:** Aaron M. Samuels, Nobert Awino, Wycliffe Odongo, Benard Abong’o, John Gimnig, Kephas Otieno, Ya Ping Shi, Vincent Were, Denise Roth Allen, Florence Were, Tony Sang, David Obor, John Williamson, Mary J. Hamel, S. Patrick Kachur, Laurence Slutsker, Kim A. Lindblade, Simon Kariuki, Meghna Desai

**Affiliations:** 1 Division of Parasitic Diseases and Malaria, Center for Global Health, Centers for Disease Control and Prevention, Atlanta, GA 30333 USA; 2 Centre for Global Health Research, Kenya Medical Research Institute, Kisumu, Kenya; 3 Centers for Disease Control and Prevention, Kisian Campus, Off Busia Road, P O Box 1578, Kisumu, 40100 Kenya

**Keywords:** Mass test and treat, Malaria, Asymptomatic infections, Transmission reduction, Kenya, Study design

## Abstract

Most human *Plasmodium* infections in western Kenya are asymptomatic and are believed to contribute importantly to malaria transmission. Elimination of asymptomatic infections requires active treatment approaches, such as mass testing and treatment (MTaT) or mass drug administration (MDA), as infected persons do not seek care for their infection. Evaluations of community-based approaches that are designed to reduce malaria transmission require careful attention to study design to ensure that important effects can be measured accurately. This manuscript describes the study design and methodology of a cluster-randomized controlled trial to evaluate a MTaT approach for malaria transmission reduction in an area of high malaria transmission. Ten health facilities in western Kenya were purposively selected for inclusion. The communities within 3 km of each health facility were divided into three clusters of approximately equal population size. Two clusters around each health facility were randomly assigned to the control arm, and one to the intervention arm. Three times per year for 2 years, after the long and short rains, and again before the long rains, teams of community health volunteers visited every household within the intervention arm, tested all consenting individuals with malaria rapid diagnostic tests, and treated all positive individuals with an effective anti-malarial. The effect of mass testing and treatment on malaria transmission was measured through population-based longitudinal cohorts, outpatient visits for clinical malaria, periodic population-based cross-sectional surveys, and entomological indices.

## Background

The threefold increase in malaria control and elimination funding over the last decade has resulted in widespread increased coverage of malaria control interventions, including vector control with long-lasting insecticidal nets (LLINs) and indoor residual spraying (IRS), improved case management with rapid diagnostic tests (RDTs) and artemisinin-based combination therapy (ACT), and intermittent preventive treatment for high-risk groups [1]. It has been postulated that the scale-up, specifically of LLINs, ACT, and IRS, is responsible for the estimated 40% decline in malaria case burden in Africa from the year 2000 to 2015 [2]. Yet, gains have been heterogeneous, and in some endemic areas, despite heavy investments in these interventions, malaria prevalence, morbidity, and mortality remain high. As more countries approach elimination or aim for rapid malaria transmission reduction towards pre-elimination, new tools and strategies are necessary to complement existing ones [3].

The search for new tools to reduce malaria transmission towards elimination has led to a renewed focus on targeting the infectious human parasite reservoir, particularly the asymptomatic population [4]. In some low transmission settings, >60% of the infected population is asymptomatic; this proportion typically increases with increasing transmission intensity [5]. Mosquito infection studies have demonstrated that blood meals from persons with asymptomatic infections can result in transmission [6–8], and it has been postulated that in areas of seasonal transmission, the asymptomatic population may sustain transmission through the dry seasons [9–11].

Additionally, individuals with asymptomatic infections are likely to be infected for longer durations of time, some for many years [12], as they are less likely to seek care and be directly impacted by strategies such as improved case management. Strategies that specifically target the asymptomatic population, such as mass test and treat (MTaT) campaigns where all community members are tested for malaria, and all positives are treated, and mass drug administration (MDA) where all community members are treated for malaria without testing, are potential complementary tools for rapid malaria transmission reduction towards elimination [13, 14].

Research trials and public health programmes implementing MDA to control or eliminate malaria have been conducted for at least a century [15, 16]. The majority of the MDA studies, including the influential Garki Project [17], have shown a significant decrease in parasite prevalence shortly after MDA rounds, but when the trials were over, and in the absence of ongoing delivery of preventive services, a rebound in infection prevalence was observed, frequently as early as 6 months after trial completion [18–22]. A recently published literature review found that only 12 of 182 (6.5%) published reports of MDA resulted in success towards elimination with a definition of zero indigenous cases of malaria in the target population for a minimum of 6 months after the end of all MDA rounds [16]. Of note, in many of these MDA trials other key malaria control interventions (high coverage with effective vector control, case management, and surveillance and response including active case detection) were not in place during or after the trials.

In the era of ACT with long chemoprophylactic half-lives (e.g., dihydroartemisinin–piperaquine) and the potential adjunct of drugs with gametocytocidal (e.g., primaquine) or insecticidal (e.g., ivermectin) activity, there is renewed interest in evaluating time-limited mass treatment strategies to accelerate transmission reduction in the context of high coverage with other malaria control interventions. Robust mathematical models have been created to explore the impact of MDAs and MTaTs on malaria transmission. These models suggest that, in conjunction with high and sustained LLIN coverage and treatment with artemisinin-based combinations, interventions targeting the asymptomatic reservoir, such as MTaT and MDA, help to drive transmission in an area towards elimination; however, results differ based on transmission setting [14, 23–28].

Additionally, recently published field studies assessing either modified focal screen and treat strategies, where small discrete geographic areas are identified for screening and treatment [29, 30], or mass test and treat [31] in different transmission settings suggest that MTaT may effectively identify the majority of the asymptomatic infected population and reduce malaria transmission. However, a study of MTaT in a low transmission setting in Zanzibar did not reduce malaria incidence [32], and more recently, and subsequent to the study described in this manuscript, a World Health Organization expert review group stated that with current diagnostic tests, MTaT could not be recommended for malaria transmission reduction [33]. Additionally, there were concerns that MDA may exert excessive drug pressure that may lead to adverse events in uninfected persons or enhance the development and spread of anti-malarial resistance; there was less concern for these negative outcomes for MTaT strategies [33, 34]. However, with MTaT, there was concern that the sensitivity of available rapid diagnostic tests would be too low to identify a large enough proportion of infected individuals to make a significant impact on malaria transition.

This manuscript describes and provides a discussion of a study design and methodology to evaluate the acceptability, effectiveness, and costing of a community-based MTaT strategy for malaria in an area of high transmission.

## Methods

### Study objectives

The objective of this study was to measure changes in malaria transmission outcomes resulting from community MTaT compared to case management alone in a setting of high coverage with LLINs. Specific primary objectives were to evaluate the:

1. Incidence of *Plasmodium falciparum* infection and clinical malaria.
2. Prevalence of *P. falciparum* infection and anaemia.
3. Sporozoite and oocyst rates in malaria vectors.

Secondary objectives included measuring the acceptability of MTaT to the local community, assessing adherence to anti-malarial regimens, costing of the intervention, and documenting the impact of MTaT on gametocyte prevalence and serologic markers of transmission.

### Study area and population

**Fig. 1:**
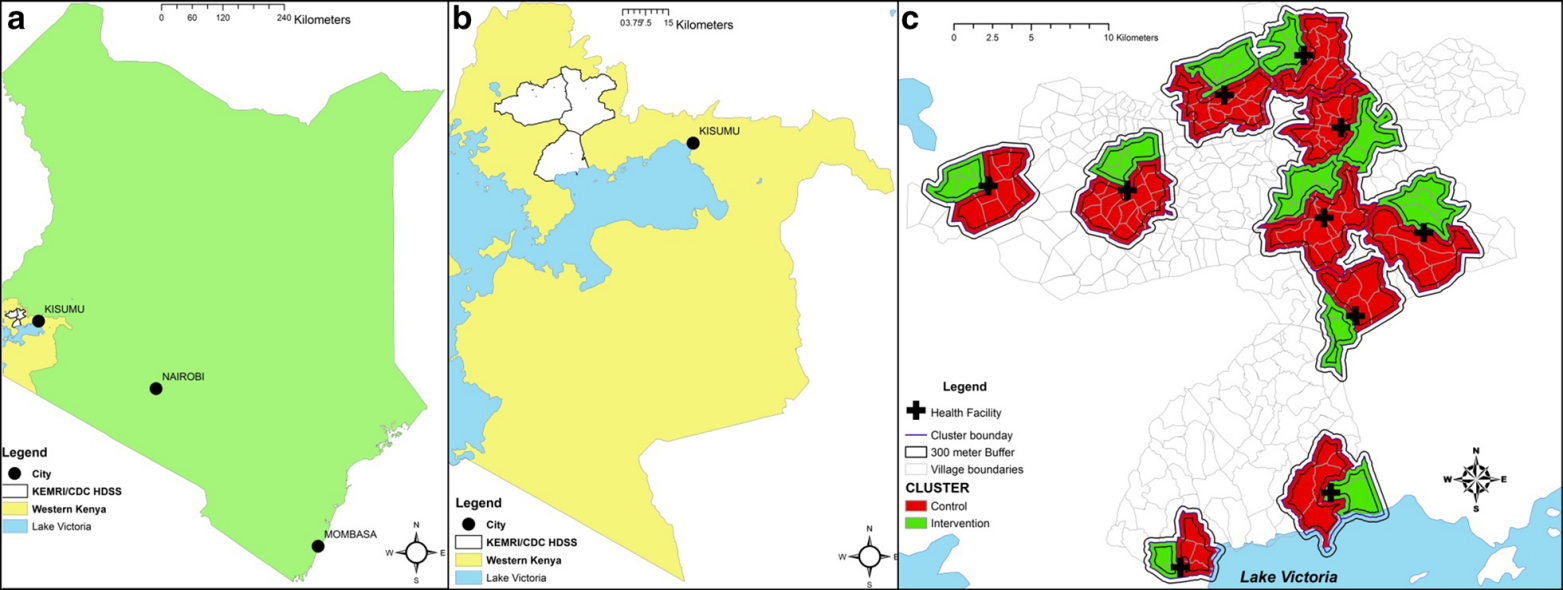
KEMRI and CDC Health and Demographic Surveillance System. **a** Location of western Kenya in Kenya. **b** Location of KEMRI and CDC Health and Demographic Surveillance System (HDSS) in western Kenya. **c** Mass test and treat clusters within KEMRI and CDC HDSS

The study was performed in Siaya County, western Kenya, within the catchment area of the Kenya Medical Research Institute (KEMRI) and Centers for Disease Control and Prevention (CDC) Health and Demographic Surveillance System (HDSS) (Fig. 1), containing approximately 220,000 individuals, described in detail elsewhere [34]. The vast majority of the HDSS population is of the Luo ethnicity, and work as subsistence farmers. Families reside in compounds that consist of the house of the head of the compound, and a house for each of his wives and their children. Three times a year, a household census is conducted in the HDSS area during which household listings are updated, compounds are mapped, and a survey instrument is administered to document household demographics including births, deaths, in-migration, out-migration, and migration from one area within the HDSS to another, termed trans-migration. To supplement information collected during HDSS survey rounds, villagers who work for the HDSS are incentivized to report births and deaths as they occur. The Ministry of Health began training and supporting Community Health Volunteers (CHVs), who themselves are community members, to implement community-based health strategies at the household level in 2013-just prior to the initiation of this study. Their activities include providing malaria-specific testing with RDTs and treatment of positives with ACT to symptomatic individuals in the household. Coverage and capacity of this strategy were low at the onset of this study.

Malaria transmission is high and occurs year-round with two peak transmission seasons in May–July and November–December, coinciding with the end of the long and short rains, respectively. LLIN distribution campaigns and the roll-out of ACT and RDTs have been supported in this area through the KEMRI and CDC collaboration and with other partners including the Kenya National Malaria Control Programme, the U.S. President’s Malaria Initiative, and the Global Fund to Fight AIDS, Tuberculosis, and Malaria. Since the widespread introduction of insecticide-treated nets into the HDSS area in 1997, malaria prevalence by microscopy has decreased in children aged 1–5 years from >70% [35] to approximately 39% in 2013; *P. falciparum* is the predominant malaria parasite species comprising >95% of all infections [36]. The proportion of community members with infections detected by microscopy who did not report a fever in the previous 2 weeks varies by age and is >90% in many age groups [Desai M, personal communication].

Since mortality data have been collected in the HDSS using verbal autopsy methods, there has been a threefold reduction in the malaria-specific mortality rate in children <5 years from 13.2 per 1000 person-years in 2003 to 3.7 per 1000 person-years in 2010 [37]. The number of malaria infective mosquito bites per night dropped from approximately 300 in the early 1990s [38] to <20 in 2010, and the vector population temporarily shifted from *Anopheles gambiae s.s.* and *Anopheles funestus* to *Anopheles arabiensis* before *An. funestus* re-emerged as the primary vector [39]. Household ownership of at least one LLIN, universal coverage (defined as 1 bed net per every two persons per household), and use prior to study initiation were 81, 67, and 57%, respectively [Were V, personal communication]. Indoor residual spraying has not been implemented as a programmatic strategy in Siaya County.

### Study design

The study was designed as a three-arm cluster-randomized controlled trial. Blinding of study participants or study staff to the intervention was not considered feasible or ethical. Ten of the 61 health facilities within the HDSS in Siaya County were purposively selected based on the following criteria: (1) high absolute malaria caseloads, (2) proximity of the facility to major roads for transport of supplies and samples, (3) government owned and run, and (4) minimum overlap with other known interventional/observational studies in the facility catchment; health facilities were chosen to maximize the distance between them, and all were at least 5 km from the next nearest health facility. All villages whose midpoint was located within 3 km of each health facility were included in the study. The villages surrounding each health facility were divided into three clusters along village boundaries such that the population was approximately evenly divided. One of the three clusters surrounding each health facility was randomly selected for the intervention, and the remaining two served as controls (Fig. 1). The purpose of the second control cluster was to allow the possible addition of a second intervention in the future. As no additional intervention arm was included in the study, for analysis purposes, the two control clusters were merged so that the study comprised 10 intervention and 10 control clusters with equal sample sizes. To reduce contamination between intervention and control clusters, the sampling frame for evaluations were limited to a core area within each cluster. The core area was defined as an area within the cluster that was ≥300 m from the perimeter of the cluster. The 300 m distance was chosen on the basis of previous studies at the site that indirectly demonstrated that the mass effect of a community based intervention (LLINs) extended to approximately 300 m based upon both entomologic [40] and epidemiologic [41] data. The area between the core and the perimeter of the cluster was defined as the buffer; though the intervention was performed in the buffer, data from individuals residing within the buffer area were not used for evaluations.

### Ethical considerations

The study protocol and informed consent and assent forms were approved by the KEMRI Scientific and Ethics Review Unit (KEMRI protocol #2380). The CDC institutional review board relied on the KEMRI Scientific and Ethics Review Unit for approval. The Kenya Pharmacy and Poisons Board approved the protocol and importation of dihydroartemisinin-piperaquine (Eurartesim^®^, Sigman-Tau, Pomezia, Italy) for the study.

### Intervention

#### Training

Prior to participant contact, study staff were trained in Good Clinical Practices and the protocol. Each cadre of staff were trained according to their study responsibilities, including consenting participants, using tablets and personal digital assistants (PDAs), administering questionnaires, collecting blood samples and performing RDTs, implementing treatment algorithms, and entering data.

#### Community mobilization

Prior to study initiation, and again before each MTaT round, local District Health Management Teams were approached to discuss the study purpose, procedures, and timelines. Formal meetings were held with the village chiefs and heads of schools within the study area. Informational letters and flyers were distributed within these communities, and were followed by *barazas* (community meetings) during which study staff briefly described the study and answered questions from the community members. Short messages were transmitted during radio-spots in the local language to broadcast to the communities, and provided information for community members to contact study staff with questions.

#### Consenting process

Prior to any interaction with a potential study member, CHVs described the study to the compound head and requested permission to individually consent all household members. Caregiver consent was required for all children <18 years of age (except mature minors, which includes pregnant women and mothers <18 years of age), and, in accordance with local guidelines, an additional written assent was required for children aged 13–17 years of age. When day-school-going children attending school and residing within the community were not available at the house, study teams obtained informed consent from the child’s parent, and then organized trips to the schools, sought permission from teachers, and identified, assented, and performed study procedures on the selected children on the school campus.

#### Community acceptance of MTaT

Two series of qualitative data collection activities were performed within six randomly selected MTaT communities prior to the intervention and after the first MTaT round to evaluate acceptability and strategies for improving the intervention. A total of 36 focus group discussions were conducted with men, women, and opinion leaders, and 12 individual or small group interviews were performed with community health workers. Methodology and results were previously reported [42].

#### Mass testing and treatment rounds

A mathematical model was calibrated to a pre-trial prevalence of 35% by slide microscopy between 2 and 10 year olds, incorporating historical trends in insecticide-treated net use and first-line treatment, as well as vector species composition and bionomics informed by entomology data from the KEMRI and CDC study site. The double-peaked seasonality pattern was generated using local rainfall data. All possible monthly combinations of a 2 year, three round per-year MTaT providing a curative drug with a prophylactic period fitted to pharmacokinetic/pharmacodynamic data of the efficacy of dihydroartemisinin–piperaquine at 80% coverage, were simulated. Correlation between rounds were simulated with a co-efficient calibrated to mimic a hard-to-reach 10% of the population who only have a 10% probability of taking the drug at each round. The optimal combination was that which minimized clinical incidence within the population over the course of the intervention. Model results suggested that three annual rounds should be performed (1) prior to the short rains, (2) after the short malaria peak, and (3) just prior to the long rains, before the major malaria transmission season (Walker P, personal communication).

**Fig. 2:**
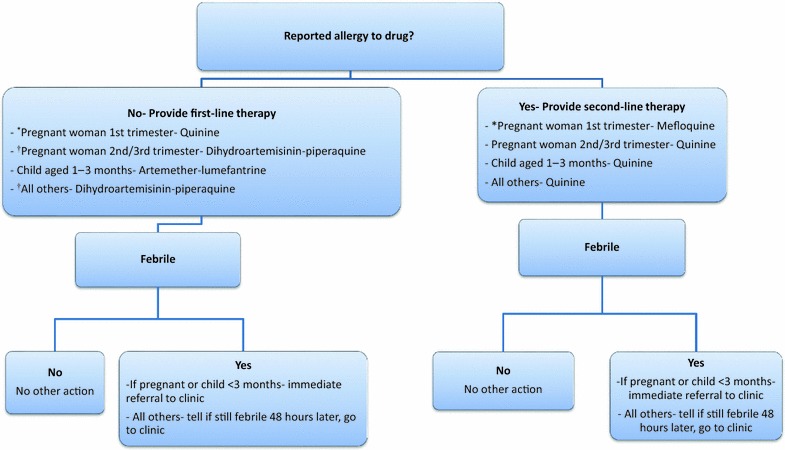
Treatment algorithm for RDT positive individuals during MTaT and XSS rounds. *Asterisk* a woman is considered potentially in her first trimester of pregnancy if she is of reproductive age (13–49 years old), is not visibly pregnant, and had her last menstrual period >4 weeks ago. ^†^During XSS rounds artemether–lumefantrine was used. *Note* During 1st and 2nd cross-sectional round all cohort members were treated with AL irrespective of RDT outcome

During MTaT rounds, CHVs visited every compound within the intervention clusters. After obtaining individual informed consent from all eligible members the CHV collected a blood sample using finger or heel prick to determine malaria parasitaemia by RDT (Carestart™ Malaria HRP-2/pLDH (Pf/PAN) Combo Test RDT; Somerset, NJ, USA). Additional drops were spotted on Whatman 903 filter papers (GE Healthcare, Marlborough, MA, USA) from the first 5000 participants encountered during the MTaT rounds to be used for real-time reverse transcriptase polymerase chain reaction (rRT-PCR) detection of malaria parasites. CHVs completed a short questionnaire programmed on PDAs using Visual CE version 12 (SYWARE^®^ Inc., Cambridge, USA) or tablets using Open Data Kit version 2.0 [43], to evaluate recent presence of malaria-associated symptoms, malaria prevention methods (e.g. LLIN use), care-seeking, use of medications, and migration. RDT positive women of child-bearing age (13–49 years of age) who were not visibly pregnant, reported a last menstrual period ≥4 weeks before the visit, and did not report being pregnant, were privately offered a urine pregnancy test at home. Those who refused pregnancy testing were referred to the closest study health facility for treatment. All participants testing positive for malaria or anaemia were treated according to an algorithm consistent with the Kenya Ministry of Health National Malaria Treatment Guidelines [44] that was pre-programmed into the PDA or tablet (Fig. 2). Those with signs of severe malaria (including prostration, altered consciousness) [44] or anaemia were referred to study health facilities for further evaluations and treatment. Study staff directly observed the first dose of anti-malarial treatment, and revisited a sample of 200 compounds, selected by probability proportional to size, to evaluate each individual who had been treated with anti-malarials one week after treatment for anti-malarial adherence. Individuals were asked how many tablets they took, and blister packs were observed for remaining tablets.

### Measurement of malaria transmission outcomes

Four separate strategies were used to evaluate outcomes related to malaria transmission: (1) annual population-based cross-sectional studies for malaria prevalence, (2) active surveillance for malaria infection utilizing a longitudinal cohort, (3) passive surveillance of laboratory-confirmed clinical malaria cases in study health facilities, and (4) entomological transmission indices. Eligible participants residing within a household located within the core area were included in the sampling frame for study evaluations and analyses. Inclusion criteria, sample sizes, and main malaria transmission outcomes for each of the evaluations can be found in Table 1.

**Table 1:** Inclusion criteria, sample size, and main outcome by evaluation type

Evaluation type	Inclusion criteria	Sample size	Main outcome
Cross-sectional studies	≥1 month of age	857 per arm	Community malaria prevalence
Longitudinal cohort study	≥1 year of age, not pregnant at time of recruitment	330 per arm	Incidence of malaria infection
Passive surveillance	Living within the core area of a cluster	N/A	Incidence of clinical malaria
Entomological surveillance	Household in either a control or intervention cluster	120 control and 60 intervention households per month	Monthly *P. falciparum* oocyst and sporozoite rates, *Anopheles* parity rate

#### Prevalence of malaria infection

Population-based cross-sectional surveys were performed annually during the peak malaria transmission season in July to estimate the point-prevalence of malaria parasitaemia and anaemia. Twenty compounds from within the core areas of each of the 20 clusters were randomly selected from the most recent HDSS housing roster for inclusion.

CHVs visited each selected compound and enrolled all residents. A questionnaire was administered to each participant, or their caregiver, which included information on demographic characteristics, risk factors for malaria infection, history of illness, health care-seeking, and costs associated with illness episodes. A blood sample was obtained using finger or heel prick to measure haemoglobin (HemoCue^®^; Ängelholm, Sweden) and malaria parasitaemia by RDT. Additionally, thick and thin blood smears were prepared for malaria microscopy and a blood spot was dried on Whatman 903 filter papers for molecular tests (Table 2). Based on RDT results, CHVs followed a treatment and referral algorithm programmed on the PDAs and tablets that evaluated the participant’s potential pregnancy status and history of allergies/adverse reactions (Fig. 2).

**Table 2:** Sample collection by evaluation method

Collection activity	Age group	Sampling	Arm	Collection location	Samples collected and diagnostics conducted
Cross-sectional surveys	≥1 month old	20 compounds from the core area per cluster	Both	Household	RDT Blood smear Dried blood spots Hemoglobin
Cohort visits (scheduled and unscheduled)	≥1 year old	Random selection from cluster cores during baseline and year 1 XSS	Both	Health facility	RDT Blood smear Dried blood spots Hemoglobin
MTaT rounds	≥1 month old	All residents	Intervention	Household	RDT Dried blood spot (from first 5000)
Passive surveillance	All ages	All individuals living within the core area of a cluster	Both	Health facility	RDT

The sample size was calculated, assuming a malaria prevalence of 40% in the control arm, a type I error rate of 5, and 80% power, to detect a relative difference in malaria prevalence of at least 50% between arms by study end. For ease of sampling, compounds were selected, and all individuals in the compounds were enrolled. A conservative coefficient of variation of 0.3 for between-cluster and—compound variance was used. Twenty compounds were selected per cluster for a total of 400 compounds across the study area (200 in each arm), and a total population of approximately 2000 individuals [45].

#### Incidence of malaria infection

The incidence of malaria infection was measured using active surveillance in a population-based cohort from the intervention and control clusters. At each cross-sectional survey, a sub-sample of individuals were recruited from both the control and intervention arms to measure the incidence of *P. falciparum* infection over time. From within the 20 compounds in each of the 20 clusters included in the cross-sectional survey, 33 individuals were randomly selected for a total sample size of 660 individuals (330 control and 330 intervention) aged ≥1 year. At the time of recruitment, all cohort participants were provided an LLIN and tested for malaria, and were treated with artemether–lumefantrine for malaria to clear subpatent infections. Cohort participants made scheduled monthly visits to the closest study health facility, and were encouraged to attend the health facility in case of illness. At both scheduled and sick visits, participants were administered a questionnaire on recent history of fever, health-seeking behaviour, LLIN use, and medication use in the month since the previous visit. Blood samples were obtained by finger pricks at each scheduled and sick visit for malaria testing by RDT and microscopy, and haemoglobin testing using a HemoCue^®^. A blood spot was also dried on a Whatman 903 filter paper for testing by rRT-PCR. Treatment decisions were made according to RDT and Hemocue^®^ results. All blood smears were read within 24 h of the visit, and participants whose blood smears were positive and discordant with the RDT result were contacted by study staff and treated.

Cohort participants not arriving for a scheduled visit were called and reminded to visit. Study staff made home visits to evaluate participants who were unreachable or who were unable to come to the clinic. Attempts to contact participants were ceased if they failed to present to three consecutive scheduled monthly visits, but they were not withdrawn from the cohort.

The sample size for the cohort study was based on detecting a relative difference in the incidence of malaria infection of at least 30% between the intervention and control arms over two years of follow-up. Based on data from previous studies, a baseline of 1.6 malaria infections per person per year, and a coefficient of variation of 0.25 were estimated for this study. A type I error rate of 5 and 80% power were used resulting in a sample size of 330 individuals per arm for a total sample size of 660 [45].

#### Incidence of clinical malaria

The incidence of clinical malaria from passive surveillance between residents of intervention and control clusters was compared. All persons registered with the HDSS and living within the core areas of clusters were considered under surveillance. Patients attending one of the 10 study health facilities were interviewed by study staff, who used laptop computers to determine if the patient was registered with the HDSS and lived in the study area. Patients not registered with the HDSS, but meeting criteria for HDSS registration [34] were asked for consent to participate, and their data were included in the study. For eligible patients, study staff recorded data from the clinical register into laptop computers, including history of fever/suspected malaria, malaria confirmatory testing and results, and medications prescribed. Per national guidelines, all patients presenting to a health facility with history of, or current fever, were considered suspected clinical malaria cases, were diagnosed by either RDT or blood smear microscopy, and were treated according to Kenya Ministry of Health Guidelines [44]. Data were retrieved from each of the 10 health facilities on a weekly basis.

#### Entomological transmission indices

Monitoring was conducted to assess the impact of MTaT on entomological indices of malaria transmission by comparing the proportion of *Anopheles* mosquitoes with *P. falciparum* sporozoite and oocyst infections, and the parity rate, by study arm.

Each month, a simple random sample of twelve and six houses were selected (without replacement) from each of the ten control and intervention clusters, respectively, for pyrethrum spray catches (PSCs). Briefly, white sheets were placed over the surface of the floor and furniture between 0600 and 1200 h, and two collectors (one inside and one outside the house) sprayed around the eaves with 0.025% pyrethrum emulsifiable concentrate with 0.1% piperonyl butoxide in kerosene. The collector inside the house then sprayed the roof and walls of the house, and the house was closed for 10–15 min. Dead mosquitoes were then collected and placed on moist filter paper inside of petri dishes and returned to the laboratory.

Live collections using Prokopack [46] aspirators were performed for one week each month in each study arm. During the live sampling week, collectors visited as many households as possible until 1200 h. To maximize the number of specimens collected, the live collection methodology did not use a random sample. In each house, collectors spent 15–20 min aspirating mosquitoes, and those collected from the same house were placed into a single cage.

#### Costing

During cross-sectional surveys, socioeconomic variables and data on household costs associated with care-seeking and treatment of febrile illnesses and malaria, including missed days of work and/or school, were collected. Socioeconomic variables were used to establish wealth quintiles. These data will be compiled to evaluate costing and differences in equity impact of the intervention. Additionally, during MTaT rounds, data for cost of the intervention per population reached were collected to evaluate cost-effectiveness and gained cost-efficiencies throughout subsequent rounds. Costs will be categorized as either intervention or research costs in order to evaluate potential programmatic delivery costs.

### Laboratory methods

#### Preparation of dried blood spots

Five blood spots were prepared, each with a minimum of 50 mm^3^ of blood, into each spot of a Whatman 903 filter paper, and were dried overnight at room temperature. Each filter paper was sealed tightly in a plastic bag with desiccants and a moisture indicator, and was transported to a central laboratory for storage at −80 °C until use.

#### Measurement of hemoglobin

Haemoglobin level was determined using portable HemoCue^®^ analyzers (HemoCue^®^ AB, Angelholm, Sweden). All HemoCue^®^ analyzers were validated through comparison to an automated Coulter Counter (Beckmann-Coulter, IN, USA) before the study and calibrated on a monthly basis using low, normal, and high hemoglobin controls supplied by the manufacturer.

#### Malaria microscopy

Thick and thin blood smears were transported to a central laboratory at Siaya County Referral Hospital daily. Blood smears were stained with 10% Giemsa, dried for 15 min, and examined for the presence of malaria parasites under oil immersion. A smear was considered negative if no parasites were found in 100 microscopic high powered fields. If positive, malaria parasites were counted against 500 white blood cells (WBC) and parasite densities were expressed per mm^3^ of blood using an assumed 8000 WBCs/mm^3^ [47–49]. All blood smears were independently read by two microscopists blinded to the other’s read, and to the participants’ study arm, who had adequately passed an external quality assurance programme provided by the National Institute of Communicable Diseases, South Africa [50]. Discordant reads, defined as reads that differed qualitatively, by species, or with a difference in parasite density of ≥50%, were confirmed by a third microscopist blinded to the results of the first two microscopists.

#### Real-time reverse transcriptase polymerase chain reaction for malaria

Real-time reverse transcriptase polymerase chain reaction was used with randomly selected RDT negative samples from the MTaT rounds to estimate the number of infections missed by RDTs. Briefly, commercially available TaqMan Universal Master Mix (Applied Biosystems, CA, USA) and species-specific probes corresponding to *P. falciparum*, *Plasmodium vivax*, *Plasmodium malariae*, and *Plasmodium ovale* were used to detect the presence of parasites. A threshold cycle number (Ct) of 40 was used as the cut-off. Similarly, randomly selected samples that were positive or negative for asexual parasites were used to determine the presence of stage V gametocytes using nucleic acid sequence based amplification (Pfs25-NASBA) assays as previously described [51].

#### Laboratory testing of mosquitoes

Mosquitoes collected during PSCs and live collections were first identified to species using morphological keys [52], while sibling species were identified by rRT-PCR for all *Anopheles gambiae s.l*. [53] and a subset of *An. funestus s.l*. [54] for the rRT-PCR identification of *An. gambiae s.l*., only the universal, the *An. gambiae s.s*. and the *An. arabiensis*, primers were used as these two species are the only ones known from this area. A subset of freshly collected, unfed mosquitoes were dissected and examined for parity status [55]. Fed, gravid and half-gravid mosquitoes were held for 5–10 days and midguts were dissected, stained with mercurochrome and then examined at 100× for the presence of oocysts. *P. falciparum* sporozoite rates in *Anopheles* mosquitoes were determined by sandwich ELISA [56, 57].

### Data analysis

The primary outcome of incidence and prevalence analyses, in the cohort and cross-sectional studies, respectively, were microscopically confirmed *P. falciparum* parasitaemia. Arm-specific unadjusted prevalence ratios (PR) for parasitaemia and anaemia were calculated using data from the series of pre- and post-intervention cross-sectional surveys. These data were analyzed with a log-binomial model using generalized estimating equations to account for clustering. Pre- and post-intervention PRs between arms were evaluated using the same model. The incidence of clinical malaria, diagnostically confirmed by either RDT or microscopy, presenting to the MTaT study health facilities (passive surveillance) was calculated. Total case counts originating from each study arm at each health facility per month was divided by the total population of the study arm residing in the catchment area of the health facility according to the HDSS. The incidence of infection was determined from the cohort data. A Cox-Proportional Hazards model was used to compare time to infection between arms. Adjusted models were created to account for confounders including socioeconomic status, anti-malarial use, and LLIN use. Socioeconomic status (SES) was assessed using principal component analysis models of household assets [58]. Sporozoite and oocyst rates were compared by logistic regression using generalized estimating equations to account for correlated observations within the households. All analyses were performed using SAS statistical software version 9.3 (SAS Institute, Carey, NC).

## Discussion

Previous results of trials or programmes incorporating mass campaigns, such as MTaT, for transmission reduction on a large-scale have been contradictory or inconclusive [31, 32]. As the population of individuals harbouring asymptomatic infections has been increasingly implicated in sustaining malaria transmission there has been renewed interest in evaluating time-limited MTaT in different transmission settings with high coverage of malaria control interventions for rapid malaria transmission reduction. The study site in western Kenya provided distinct advantages for evaluating MTaT including; (1) high malaria transmission despite high coverage with LLINs, (2) the existence of a HDSS through which sampling frames and population level data including births, deaths, and migration have been continuously monitored, (3) over 30 years of historical data on epidemiological and entomological malaria indices, (4) advanced laboratories and research infrastructure, and (5) strong relations with the communities and public and private health sectors in the region. In this manuscript, the strengths and limitations of the study design choices are discussed.

Interventions such as MTaT are designed to be effective at high coverage levels and, therefore, must be evaluated at the community level, but the cost and complexity of cluster-randomized trials combined with the heterogeneity of malaria transmission and differential access to health care complicate the design of such trials. To minimize differences in the spatial distribution of malaria transmission, differences in treatment availability and provider proficiency, the effect of distance on health-care seeking, and LLIN coverage, each cluster around the study health facilities was randomly assigned to the intervention or control arm, and only villages with a centroid within 3 kms from the health facilities were included. The result of this decision was that clusters abutted one another and were relatively small for campaign-style interventions, which are intended to be implemented over larger geographic spans for transmission reduction. This smaller size and geographic proximity may artificially increase the impact of migration as individuals may be more likely to migrate out of a smaller geographic cluster than a larger one, and through daily movement, may be more likely to enter a cluster that is closer to where they reside than one that is geographically distant.

Migration is known to play a major role in malaria transmission as parasites are transported from one area to another [59], or one cluster to another. In this trial, migration and contamination of parasites into other clusters, was likely non-differential, and thus may bias the outcome to the null. It was recognized that human migration was unavoidable and would serve as a limitation to evaluation, and measures were taken to quantify it during MTaT rounds, at cohort visits, and through the HDSS census, however daily movement between clusters was not quantified. To mitigate the impact of daily movement on the outcome measure while maintaining statistical tenets for cluster numbers, there was a consideration for creating much larger clusters that were geographically distant from one another, while maintaining the same number of clusters for statistical validity. However, this would have been cost-prohibitive and the necessary geographic separation would likely have led to significant confounding from transmission differences and health facility associated factors such as stockouts and care-seeking behaviour. When choosing a study design, researchers must carefully weigh the benefits of bias mitigating decisions versus the introduction of contamination. Smaller clusters were used in this study. Other study designs for evaluating population-based interventions such as stepped-wedge approaches, through which larger clusters can be more easily created, were considered, however at the time, the statistical rigor and the practicality of these designs were in question [60, 61].

The frequency and timing of test and treat rounds in this study are based on robust mathematical models. These models account for multiple variables including LLIN coverage, usage, and baseline transmission levels. All models are based on assumptions from years of research in malaria in general, and specifically to the study site. The models suggest that clearing the parasite reservoir during the dry season, when the number of sub-clinical infections and vectors are lowest, would be the most effective way of reducing transmission, and that spacing the rounds 1–2 months from each other and ensuring at least one of the rounds is delivered immediately before the peak transmission season would be the most cost-effective way of performing this. However, model assumptions are constantly being updated as new field data are collected. Data from this study will be incorporated into the models to refine them.

## Conclusions

Ultimately, the success of MTaT campaigns in rapidly reducing malaria transmission or eliminating malaria will reside on community acceptability, the degree and duration of impact, number of years of intervention needed, and the cost-effectiveness. Data from the implementation of this study will be used to inform policy and to refine mathematical models for more precise estimates in high transmission settings.

## Abbreviations

LLIN: Long-lasting insecticide-treated net
IRS: Indoor residual spraying
ACT: Artemisinin-based combination therapy
RDT: Rapid diagnostic test
MTaT: Mass testing and treatment
MDA: Mass drug administration
KEMRI: Kenya Medical Research Institute
CDC: Centers for Disease Control and Prevention
HDSS: Health and demographic surveillance system
CHV: Community health volunteer
XSS: Cross-sectional study
PDA: Personal digital assistant
CO: Clinical officer
rRT-PCR: Real-time reverse transcriptase polymerase chain reaction
PSC: Pyrethroid spray catch
PR: Prevalence ratio
